# Impacts of preoperative anxiety and depression on pain and range of motion after arthroscopic frozen shoulder release: a cohort study

**DOI:** 10.1007/s00264-024-06186-5

**Published:** 2024-04-25

**Authors:** Yahia Haroun, Ahmed Saeed Younis, Wessam Fakhery Ebied, Mohamed Amr Hemida, Ahmed H. Khater

**Affiliations:** 1 Orthopedic Department, Faculty of Medicine, Ain Shams University, Cairo, Egypt; 2 Lecturer of Orthopedic Surgery, Ain Shams University, Cairo, Egypt; 3 Associate Professor of Orthopedic Surgery, Ain Shams University, Cairo, Egypt

**Keywords:** Frozen shoulder release, Pain, Motion, Hospital anxiety depression scale, HADS

## Abstract

**Purpose:**

We aimed to evaluate the impact of preoperative anxiety and depression levels on baseline and postoperative pain in patients who underwent arthroscopic frozen shoulder release.

**Methods:**

The study included 59 patients with more than three months of idiopathic frozen shoulder. All patients had arthroscopic frozen shoulder release. Two patients were excluded from statistical analysis. Therefore, the statistical analysis was performed on the remaining 57 patients. The patients were divided into two groups according to HADS scores: group 1 which included 28 patients with a healthy psychological status (anxiety ≤ 7 and depression ≤ 7), and Group 2, which included 29 patients with psychological distress ( anxiety ≥ 8 or depression ≥ 8).

**Results:**

The hallmark finding of this study is that patients complaining of frozen shoulder symptoms and having psychological distress (HADS ≥ 8) experienced higher pain scores preoperatively and at one-year follow-up after arthroscopic release. All patients showed significant improvement between the preoperative period and the one year follow-up regarding the abduction, forward flexion, external rotation at the side and the VAS pain score with a *P* value of 0.001.

**Conclusions:**

Arthroscopic frozen shoulder release significantly lowers the VAS pain score over the 12-month.

## Introduction

Frozen shoulder is a debilitating condition that can severely restrict the activities of daily living due to the excruciating pain and limited range of motion [1]. Arthroscopic capsular release is a safe and fast solution that offers long-lasting improvement of shoulder function when conservative treatment fails [2]. The association between chronic musculoskeletal pain and depression/anxiety leads to more severe pain and greater impairment [3, 4]. Attention should be given to the patient's psychological state as it may have a negative impact on the outcome of shoulder surgery. Orthopaedic surgeons should plan the best management approach accordingly [5]. Anxiety and depressive symptoms are commonly reported using tools such as the Hospital Anxiety and Depression Scale (HADS). The HADS consists of two 7-item subscales measuring anxiety (HADS-A) and depression (HADS-D) [6]. Due to its high consistency and reliability, it is commonly used as a screening tool to detect anxiety and depression in patients with musculoskeletal disorders [6, 7]. Each item on the questionnaire is rated on a scale of 0 to 3. The final score for anxiety and depression ranges from 0 to 21. Higher scores indicate a greater likelihood of anxiety or depression. An optimal balance between sensitivity and specificity was achieved by using a cutoff score of ≥ 8 for both the HADS-A and the HADS-D [8]. To our knowledge, there is a scarcity of literature that thoroughly examines the relationship between psychological distress and functional baseline scores in frozen shoulder [3, 9]. We aimed to evaluate the impact of preoperative anxiety and depression levels on baseline and postoperative pain in patients who underwent arthroscopic frozen shoulder release.

## Methodology

We conducted a prospective cohort study at the University Hospitals between November 2022 and December 2023. Following the University ethical committee approval, we included a consecutive of 59 patients who presented to the outpatient specialized shoulder clinic with a diagnosis of idiopathic frozen shoulder. All patients had shoulder pain and limited shoulder motion for more than three months with failure of conservative treatment. All patients were initially treated with a combined protocol of analgesics, intra-articular steroid injection and physiotherapy [10, 11]. The diagnosis was based on history and clinical examination. Shoulder magnetic resonance imaging (MRI) was done for all patients to rule out any other intra-articular shoulder pathology. We excluded patients with glenohumeral arthritis, neural damage, rotator cuff tear, previous shoulder surgery or fracture and secondary frozen shoulder. All participants signed the informed consent. We collected the demographic data for each patient -which included age, sex, medical history, duration of symptoms and the affected side-and measured shoulder range of motion using a goniometer. Range of motion included forward flexion, external rotation at the side, and abduction. All patients filled out a visual analogue scale for pain (VAS) (0, no pain; 10, most severe pain) and HADS [12, 13]. The patients were divided into two groups according to HADS scores: group 1, those with a healthy psychological status (anxiety ≤ 7 and depression ≤ 7), and Group 2, those with psychological distress (anxiety ≥ 8 or depression ≥ 8).

### Operative technique

All arthroscopic procedures were performed by a single senior operator, All patients received general anaesthesia and a beach chair position was utilized in all patients of the study group. Standard shoulder arthroscopy using anterior and posterior portals was done with an inspection of the articular surface of the rotator cuff and biceps tendon. All patients underwent 360° release of the anterior and posterior capsules using a radiofrequency probe, biceps tenotomy by cutting the long head of biceps tendon and shoulder mobilization.

All patients started a supervised rehabilitation program on the same day of surgery with active range of motion and mobilization. All patients began the same rehabilitation program to maintain the range of motion gained intraoperative and enhance the return to daily activities. Rehabilitation program involved sessions of aggressive passive and active assisted range of motion, stretch exercises and rotator cuff strengthening for ten weeks [14]. On discharge all patients were prescribed painkillers on demand, patients diagnosed as having anxiety and depressive disorder were referred to the psychiatric clinic to receive treatment according to the case.

### Follow-up strategy and outcomes

All patients came to routine regular follow-up in the outpatient specialized shoulder clinic, after two weeks for stitch removal and subsequent follow-ups to examine for clinical improvement and exclude any operative-related complications.

At the annual follow-up, a blinded outcome assessor collected the postoperative data of each patient. Postoperative data included the numeric visual analogue scale (VAS) (0–10) for pain [15] and shoulder range of motion. Range of motion included forward flexion, external rotation at the side, and abduction.

## Statistical analysis

Collected data was stored in **Microsoft® Excel® for Microsoft 365 MSO (Version 2302 Build** 16.0.16130.20848) 64-bit and analyzed using IBM® SPSS® statistics version 23 metric Data was subdivided to parametric and non-parametric data using Shapiro and Kolgomorov tests [16].

Data was expressed as Mean ± SD for quantitative measures and in both number and percentage for categorized data, median and range were used in reporting HADS and VAS pain score. The following tests were done: 1. Comparison between two independent mean groups for parametric data using paired t-test. 2. Comparison between two independent groups for non-parametric data using Mann–Whitney U test. 3. Chi-square test to study the association between each two variables or comparison between two independent groups as regards the categorized data. 4. comparison between two dependent groups for non-parametric data using the Wilcoxon Signed Ranks test. The probability of error at 0.05 was considered significant.

## Results

This study included 59 adult patients, who underwent arthroscopic frozen shoulder release for the treatment of idiopathic frozen shoulder of at least three months duration with failed conservative management. Out of the total 59 patients who underwent routine OPC follow-ups, two patients were excluded from the statistical analysis. One patient had a recurrence of frozen shoulder symptoms at the four month interval follow-up and received an intra-articular steroid injection and mobilization under general anaesthesia, while the other patient was excluded as he missed the follow-ups. The final analyses included 57 patients divided into two groups. Group 1 which included 28 patients with a healthy psychological status (anxiety ≤ 7 and depression ≤ 7), and Group 2, which included 29 patients with psychological distress (anxiety ≥ 8 or depression ≥ 8). Only one patient on group two already had a diagnosis of a major depressive disorder on selective serotonin reuptake inhibitor (SSRI) Fluoxetine, but the other patients in group 2 were newly diagnosed with psychological distress. Study population included 35 (61.4%) females and 22 (38.6%) males, the age range was from 34 to 65 years with a mean of 50.18 ± 7.96 years. 29 patients had right-sided frozen shoulder, while 28 patients had left-sided frozen shoulder. All patients had symptoms for more than three months with a range of symptoms duration from four to 24 months (Table 1).

**Table 1:** Comparison between groups regarding demographic data and characteristics of the studied patients

		Group 1	Group 2	*P*-value
		No. = 28	No. = 29	
Age at surgery	Mean ± SD	48.54 ± 7.62	51.76 ± 8.10	0.128
	Range	36 – 63	34 – 65	
Sex	Female	17 (60.7%)	18 (62.1%)	0.916
	Male	11 (39.3%)	11 (37.9%)	
MEDICAL History	Free	14 (50.0%)	11 (37.9%)	0.359
	DM	12 (42.9%)	16 (55.2%)	0.352
	HTN	2 (7.1%)	1 (3.4%)	0.532
	DM Cardiac stent	0 (0.0%)	2 (6.9%)	0.157
	Hypothyroid	1 (3.6%)	0 (0.0%)	0.305
	Thyroid	2 (7.1%)	0 (0.0%)	0.143
Side	RT	13 (46.4%)	16 (55.2%)	0.509
	LT	15 (53.6%)	13 (44.8%)	
Duration of symptoms in months	Mean ± SD	7.71 ± 2.26	9.10 ± 3.61	0.088
	Range	4 – 12	5 – 24	

After assessment of HADS score for all patients. Thirty (52.6%) patients had a normal HADS A score (0–7), nine (15.7%) patients had a score of (8- 10) and eighteen(31.5%) patients had a score of (11–21). 29(50.8%) patients had a HADS D score of (0–7), thirteen (22.8%) patients had a score of (8–10) and fifteen (26.3%) patients had a score of (11–21) (Table 2).

**Table 2:** Baseline HADS score for all patients

	HADS A score			HADS D score			Overall HADS score	
	Normal (0–7)	Borderline (8–10)	High (11–21)	Normal (0–7)	Borderline (8–10)	High (11–21)	Normal	Abnormal
No. of patients (percentage)	**30 (52.6%)**	**9 (15.7%)**	**18 (31.5%)**	**29(50.8%)**	**13 (22.8%)**	**15(26.3%)**	**28 (49.1%)**	**29 (50.9%)**

The two groups were comparable regarding age, sex, medical history and comorbidities, sidedness, and duration of symptoms (Table 1). All patients showed significant improvement in the abduction, forward flexion and external rotation at the side between the preoperative period and the one year follow-up (Table 3).

**Table 3:** Range of motion (ROM) and VAS score pre and postoperative among the studied patients

		Preoperative	Postoperative	Difference	*P*-value
Abd	Mean ± SD	47.19 ± 21.44	174.56 ± 4.73	127.37 ± 20.58	< 0.001
	Range	5 – 86	160 – 180	91 – 175	
FF ROM	Mean ± SD	47.12 ± 21.38	174.44 ± 4.83	127.32 ± 22.14	< 0.001
	Range	5 – 90	162 – 180	80 – 170	
ER side	Median (IQR)	10 (8 – 20)	46 (45 – 52)	35 (27 – 45)	< 0.001
	Range	0 – 43	38 – 72	9 – 65	
VAS	Median (IQR)	7 (5 – 9)	0 (0 – 1)	-6 (-8 – -5)	< 0.001
	Range	1 – 10	0 – 3	-10 – 0	

The mean postoperative abduction range was 175.64 (4.63) in group 1 and 173.18 (4.46) in group 2 with a statistically significant difference. Forward flexion and external rotation showed no significant difference between groups (Table 4).

**Table 4:** Mean postoperative range of motion in both groups

Postoperative abduction group 1 Mean(SD)	Postoperative abduction group 2 Mean(SD)	*P* value
175.64 (4.63)	173.18 (4.46)	0.05
Postoperative forward flexion group 1 Mean(SD)	Postoperative forward flexion group 2 Mean(SD)	
174.85 (4.69)	173.54 (4.99)	0.32
Postoperative external rotation group 1 Mean(SD)	Postoperative external rotation group 2 Mean(SD)	
49.14 (8.59)	49.22 (8.59)	0.96

The median preoperative VAS score was 7 (1–10), this score showed significant improvement in the 12 months post-operative follow-up and decreased to a range of zero to 3, with a median value of zero with a *P* value of < 0.001.

**Fig. 1:**
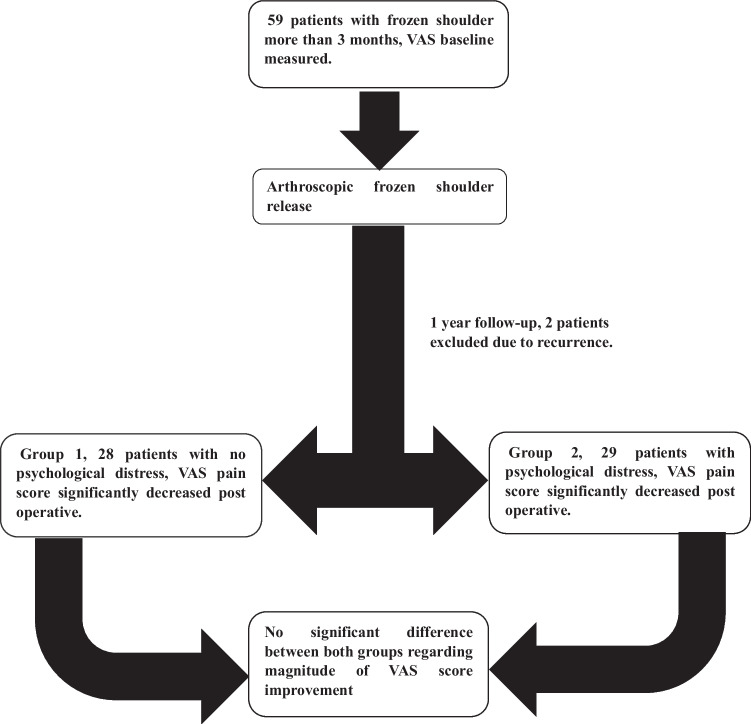
Clinical trial flowchart

When comparing the 2 groups, Preoperative and postoperative VAS score was significantly better in group 1 (patients with no psychological distress and HADS score < 8) vs group 2 (patients with psychological distress and HADS score ≥ 8). Both groups showed a significant drop in the VAS pain score between the preoperative assessment and the one year post-operative follow-up. Upon comparing the degree of pain score improvement between the two groups, there was no statistically significant difference. This indicates that both groups improved equally, with a median drop in the VAS score value of 5.5 in group 1 and 7 in group 2 (Fig. 1) (Table 5).

**Table 5:** Difference in VAS score between groups

		Group 1 No. = 28	Group 2 No. = 29	P-value
Baseline VAS score	Median (IQR) Range	6 (5 – 7) 3 – 10	8 (7 – 9) 1 – 10	0.003
Post-operative VAS score	Median (IQR) Range	0 (0 – 1) 0 – 2	1 (0 – 1) 0–3	0.039
Difference in VAS (Degree of improvement)	Median (IQR) Range	-5.5 (-7 – -4.5) -10 – -3	-7 (-9 – -6) -10 – 0	0.069

## Discussion

The relation between chronic musculoskeletal pain (CMP) and patients psychological status have been widely studied. CMP negatively affects patients mood, behaviour and psychological status [17, 18]. Also patients with psychological distress are at increased risk to experience chronic pain in a complex interplay. Studies showed that pain perception differs between individuals depending on cognitive and emotional factors [19]. Also studies showed that musculoskeletal pain perception is higher in diabetic women who have a higher prevalence of adhesive capsulitis [20–22].

Anxiety and depression have a high negative impact on general health and quality of life. Despite the recent awareness of the problem, high percentage of this disorders are undiagnosed and untreated [23, 24]. This study investigated the effects of anxiety and depression, as measured by the Hospital Anxiety and Depression Scale on clinical outcomes following arthroscopic frozen shoulder release. The hallmark finding of this study is that patients complaining of frozen shoulder symptoms and having psychological distress (HADS ≥ 8) experienced higher pain scores preoperatively and at one-year follow-up after arthroscopic release.

We studied 57 patients with more than three months duration of idiopathic frozen shoulder with a mean age of 50.18 ± 7.96. 29, 35 (61.4%) females and 22 (38.6%) males. All patients showed significant improvement between the preoperative period and the one year follow-up regarding the abduction, forward flexion, external rotation at the side and the VAS pain score. Patients with no psychological distress sowed a significant improvement in abduction range.

Similarly, in 2023 Galasso et al. studied 78 patients with a diagnosis of frozen shoulder, they reported statistically significant improvement in ROM and numerical rating scale for subjective measurement of pain, between the preoperative and postoperative periods [25]. When comparing the two groups, it was found that the Preoperative and one year postoperative VAS score was significantly higher in Group 2. Likewise, in 2019, Ebrahimzadeh et al. studied 120 patients with idiopathic frozen shoulder and concluded that the baseline VAS pain score was significantly higher in patients with anxiety and in patients with depression, but they didn’t assess the VAS pain score postoperatively [26]. Regarding the postoperative VAS pain score, similar results were reported by Park et al. in 2023. They studied 144 patients who underwent arthroscopic repair for rotator cuff tear. They reported that at six months postoperatively, the mean VAS score was still significantly lower in patients with healthy psychological states than in patients with abnormal psychological states (0.8 ± 1.6 vs 1.8 ± 2.1, respectively). However, they compared the VAS score between the two groups up to the 6-month follow-up [27]. In a prospective study, Ding et al. evaluated the impact of anxiety and depression on patients with frozen shoulder. Their findings indicated that patients who exhibited symptoms of anxiety and depression experienced more pain and upper limb disabilities than patients who did not exhibit those symptoms [28]. In the current study, both groups experienced a significant decrease in the VAS pain score between the initial evaluation before the surgery and the follow-up evaluation conducted one year after the surgery. When the degree of improvement in pain score was compared between the two groups, there was no statistically significant difference. This implies that both groups showed equal improvement, with a drop in the median value of the VAS score by 5.5 in group 1 and 7 in group 2.

The evaluation of the impact of the baseline abnormal psychological status on the degree of improvement in subjective pain perception was evaluated by the VAS pain score is underexplored in the literature. This study conclusively demonstrates the direct correlation between anxiety, depression and postoperative pain following arthroscopic frozen shoulder release. Our results showed that arthroscopic release of idiopathic frozen shoulder offers equal benefit in pain relief to patients with and without psychological distress, however, patients with anxiety or depression, still have significantly higher pain scores at least for the first post-operative year.

The findings are a critical step forward in the field, paving the way for future research to investigate the efficacy of implementing psychological interventions during the perioperative period. The limitations to the current study were that anxiety and depression were assessed only through a self-rated questionnaire and not confirmed through a diagnostic examination by a psychiatrist. This made a possibility of response bias and can lead to a misinterpretation of the presence of anxiety and depression. Also, anxiety and depression were only assessed preoperatively, not postoperatively, the interval changes in HADS-A and HADS-D scores could not be estimated.

## Conclusion

Arthroscopic frozen shoulder release significantly lowers the VAS pain score over the 12-month follow-up. Patients with established anxiety or depression have significantly higher pain scores both in the preoperative and the 12-month post-operative follow-up, this highlights the importance of addressing and managing the patients' concerns and anxiety and referral to a specialized psychiatrist or more directed rehabilitation protocol whenever needed. The magnitude of improvement in the VAS pain score in patients with anxiety or depression is the same as in patients without anxiety or depression.

## Data Availability

All data and material are available when required.

## Code availability

All software application and custom codes are available when needed.
